# Three‐dimensional imaging of the human internal acoustic canal and arachnoid cistern: a synchrotron study with clinical implications

**DOI:** 10.1111/joa.12926

**Published:** 2018-12-18

**Authors:** Xueshuang Mei, Nadine Schart‐Morén, Hao Li, Hanif M. Ladak, Sumit Agrawal, Robert Behr, Helge Rask‐Andersen

**Affiliations:** ^1^ Department of Surgical Sciences Section of Otolaryngology Uppsala University Hospital Uppsala Sweden; ^2^ Department of Otolaryngology Peking University Shenzhen Hospital Shenzhen China; ^3^ Department of Otolaryngology‐Head and Neck Surgery Department of Medical Biophysics Department of Electrical and Computer Engineering Western University London ON Canada; ^4^ Department of Otolaryngology‐Head and Neck Surgery Western University London ON Canada; ^5^ Department of Neurosurgery and Outpatient Clinic Klinikum Fulda Academic Hospital of University of Marburg Marburg Germany

**Keywords:** human, micro‐computerized tomography, synchrotron phase contrast imaging, temporal bone, Uppsala collection

## Abstract

A thorough knowledge of the gross and micro‐anatomy of the human internal acoustic canal (IAC) is essential in vestibular schwannoma removal, cochlear implantation (CI) surgery, vestibular nerve section, and decompression procedures. Here, we analyzed the acoustic‐facial cistern of the human IAC, including nerves and anastomoses using synchrotron phase contrast imaging (SR‐PCI). A total of 26 fresh human temporal bones underwent SR‐PCI. Data were processed using volume‐rendering software to create three‐dimensional (3D) reconstructions allowing soft tissue analyses, orthogonal sectioning, and cropping. A scalar opacity mapping tool was used to enhance tissue surface borders, and anatomical structures were color‐labeled for improved 3D comprehension of the soft tissues. SR‐PCI reproduced, for the first time, the variable 3D anatomy of the human IAC, including cranial nerve complexes, anastomoses, and arachnoid membrane invagination (acoustic‐facial cistern; an extension of the cerebellopontine cistern) in unprocessed, un‐decalcified specimens. An unrecognized system of arachnoid pillars and trabeculae was found to extend between the arachnoid and cranial nerves. We confirmed earlier findings that intra‐meatal vestibular schwannoma may grow unseparated from adjacent nerves without duplication of the arachnoid layers. The arachnoid pillars may support and stabilize cranial nerves in the IAC and could also play a role in local fluid hydrodynamics.

## Introduction

Vestibular nerve schwannoma removal and cochlear implantation (CI), as well as other surgical interventions such as facial nerve (FN) decompression and vestibular nerve section, require a detailed comprehension of the complex anatomy of the internal acoustic canal (IAC). Proper identification of various nerve components is essential in patients with congenital malformations (Sennaroglu, 2016). There has been debate about the extension and relationship of the cranial nerves to the acoustic‐facial cistern, which is relevant to vestibular schwannoma surgery (Yasargil et al. 1977; Lescanne et al. 2002). Also, the location and configuration of the extensive nerve connections in the IAC need further exploration.

The intricate nerve anatomy with numerous connections in the IAC was studied by several investigators in early anatomic works, using different techniques and species (Retzius, 1881; Streeter, 1906; Voit, 1907; Oort, 1918; de No, 1926; Hardy, 1934; Paturet, 1951; Shute, 1951; Gacek & Rasmussen, 1961). The studies were followed by clinically oriented investigations (House, 1961; Rhoton et al. 1968; Fisch, 1973; Chouard, 1975; Silverstein, 1984; Silverstein et al. 1988; Ryu et al. 1999; Agirdir et al. 2001; Kos et al. 2006) including micro‐dissections (Bergstrom, 1973), descriptions of meningeal layers (Lescanne et al. 2002), magnetic resonance imaging (MRI; Rubinstein et al. 1996; Unel et al. 2012), and electron microscopy combined with cadaveric anatomy (Arnesen, 1984; Ozdogmus et al. 2004).

Recently, we performed a three‐dimensional (3D) analysis of the fundus of the human IAC, using micro‐computed tomography (CT; Schart‐Moren et al. 2017a). Reconstructions displayed anatomic variations of the bony labyrinth and IAC nerve foramina, but we could not reproduce soft tissue in this study. These anatomic variations and the limited resolution of current imaging techniques motivate further exploration of reproduction of this clinically important region. Here, we describe the use of non‐invasive X‐ray imaging techniques to trace and visualize the soft tissue contents of the human IAC. High‐resolution synchrotron radiation phase contrast imaging (SR‐PCI) with 3D renderings of un‐decalcified human temporal bones was used. This allowed the tracing of separate nerve complexes, anastomoses, connective tissue sheets, and blood vessels. The acoustic‐facial cistern, an arachnoid layer including a trabecular or pillar network, could be well delineated. These arachnoid pillars or villi may support and stabilize cranial nerves in the IAC and could play a role in local fluid hydrodynamics.

## Materials and methods

The SR‐PCI technique used was recently described by Elfarnawany et al. (2017) and Koch et al. (2017). A total of 26 fresh‐frozen, then fixed, adult cadaveric temporal bones were used in this study. All specimens were obtained with permission from the body bequeathal program at Western University, London, Ontario, Canada, in accordance with the Anatomy Act of Ontario and Western University's Committee for Cadaveric Use in Research (#19062014 ‘Imaging of the Cochlea’). After thawing, a cylindrical cutter was used to core a sample (40 mm diameter, 60 mm length) from the middle ear of each temporal bone. The samples were fixed in a 4F1G bath (3.7% formaldehyde and 1% glutaraldehyde in phosphate buffer) for 5 days. The samples were rinsed twice and dehydrated using an ethanol series (50, 60, 70, 80, 90, 95, and 100%). No additional processing (i.e. staining, sectioning or decalcification) was performed on the samples. Sample fixation eliminated the risk of degradation over the 2‐month time difference between imaging sessions and scanning. Samples were transferred to the imaging facilities in motion‐proof containers to prevent damage during shipping.

The PCI technique was in‐line PCI, which has a setup similar to conventional radiography. It consists of an X‐ray source, a sample, and a detector with no other optical elements. The detector is placed at a distance from the sample which allows the phase‐shifted beam to interfere with the original beam and produce measurable fringes. The fringes correspond to the surfaces and structural boundaries of the sample (edge enhancement), compared with a conventional radiogram, which does not. To obtain SR‐PCI images, each sample was scanned using the BMIT 05ID‐2 beamline at Canadian Light Source Inc. in Saskatoon, SK, Canada. This device provides an SR beam produced by a superconducting wiggler source (Wysokinski et al. 2015). The beam is filtered, using a monochromator, and yields an energy bandwidth of Δ*E*/*E* = 10^−3^ over an energy range of 20–150 keV (Elfarnawany et al. 2017). The imaging setup, installed at a beamline length of 55 m from the source, consists of a sample stage and a charge‐coupled device‐based detector system, which are both placed on a vibration isolation table. The distance between the sample and detector was 2 m, and the photon energy was 47 keV. Motorized alignment stages were used to align the sample and detector for high‐resolution tomography. The detector was an AA‐60 beam monitor coupled with a C9300‐124 camera (Hamamatsu Photonics, Shizuoka, Japan) which has a 12‐bit resolution and an effective pixel size of 9 × 9 μm^2^. The imaging field of view was set to 4000 × 950 pixels, corresponding to 36.0 × 8.6 mm; 3000 projections over 180 rotations were acquired per view. The 3D image volume had an isotropic voxel size of 9 μm. The acquisition time to capture all projections per view was ~ 30 min. Whereas CT imaging is absorption contrast‐based, PCI can potentially be combined with synchrotron imaging to improve soft‐tissue contrast while maintaining accurate visualization of bone. Conventional absorption contrast‐based CT depends on the attenuation of X‐rays, whereas in PCI, the phase shift caused by the sample is transformed into detectable variations in X‐ray intensity. PCI can provide edge enhancement by emphasizing the contrast between boundaries of different structures in the image. The results demonstrate that SR‐PCI can be used to visualize both bone and soft tissue simultaneously.

Histological sections of the human IAC were kindly provided by Dr. Charles Liebermann at the Massachusetts Eye and Ear Infirmary, Boston, MA, USA. Hematoxylin and eosin (HE)‐stained celloidin sections were scanned, and digitized Poschl reformats were collected.

## Results

Dissections of the temporal bone occasionally injured the soft tissue in the IAC. Three cadaver bones showed comprehensive preservation of the entire IAC, which prevented more extensive quantitative evaluation of the anatomical structures. Pseudo‐colorization and adjustment of scalar opacity mapping were used to enhance surface borders and improve contrast separation and 3D conception.

The 3D imaging allowed visualization of the cochleo‐vestibulo‐facial nerve complex but not the vestibular ganglia located in the nerves (ganglion Scarpa). The cochleo‐vestibular nerve (CVN) divided into the cochlear and vestibular branches near the mid‐portion of the IAC. The last part to separate was that between the inferior vestibular nerve (IVN) and the high‐frequency acoustic nerve fibers. These connections could be demonstrated using orthogonal sectioning from various angles.

**Figure 1:**
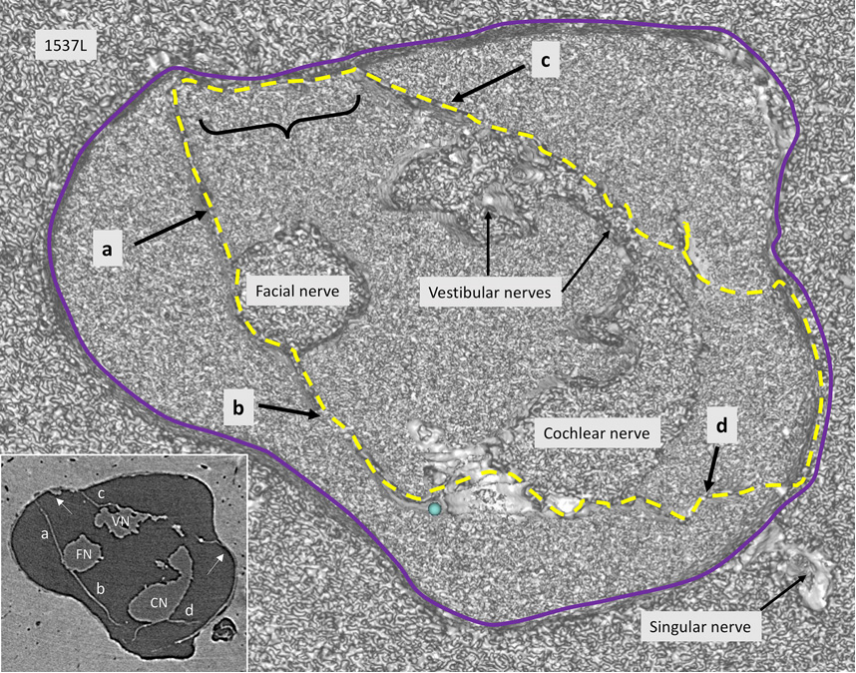
SR‐PCI of a transversely cropped right‐sided IAC near the fundus, using composite with shading technique. The arachnoid membranes (interrupted line) surround the cranial nerves. In one place (curly bracket), the arachnoid adheres to the bony wall of the IAC. Inset shows a more lateral section, verifying that the membrane is tagged (arrows) to the dura wall (blue line).

**Figure 2:**
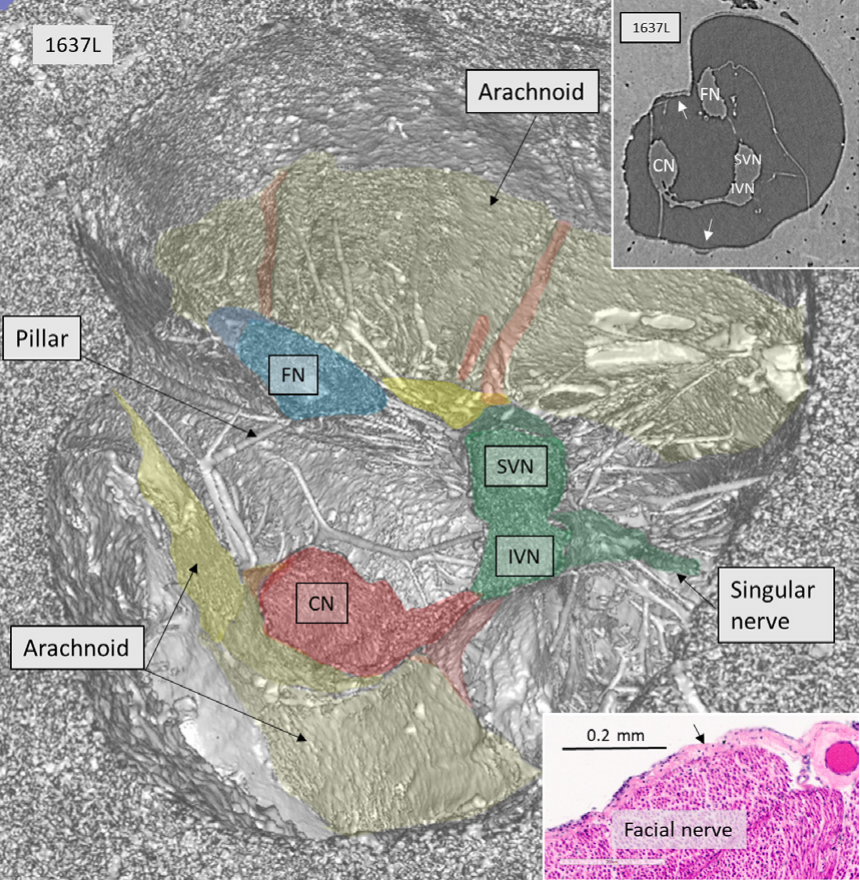
SR‐PCI, 3D reconstruction, and section near the fundus of a right human IAC. The vestibulo‐cochlear complex has separated into the auditory (red) and vestibular (green) nerve branches. There is a small connection between the nerves. In this region, the SiN supplying the posterior ampule is seen. The vestibular nerve divides into the SVN and IVN. The arachnoid membranes forming the medial and superior wall of the cistern are seen. Some vessels run in the membrane at the roof. There are several pillars between the nerves and the wall of the IAC. Upper inset shows X‐ray section at the corresponding level. The lateral wall of the cistern has merged with the IAC bony surface (arrows). Lower inset shows histological H&E‐stained celloidin section of the FN with attached arachnoid (arrow). Histological section was kindly provided by Dr. Charles Liebermann, Massachusetts Eye and Ear Infirmary, Boston, MA, USA.

**Figure 3:**
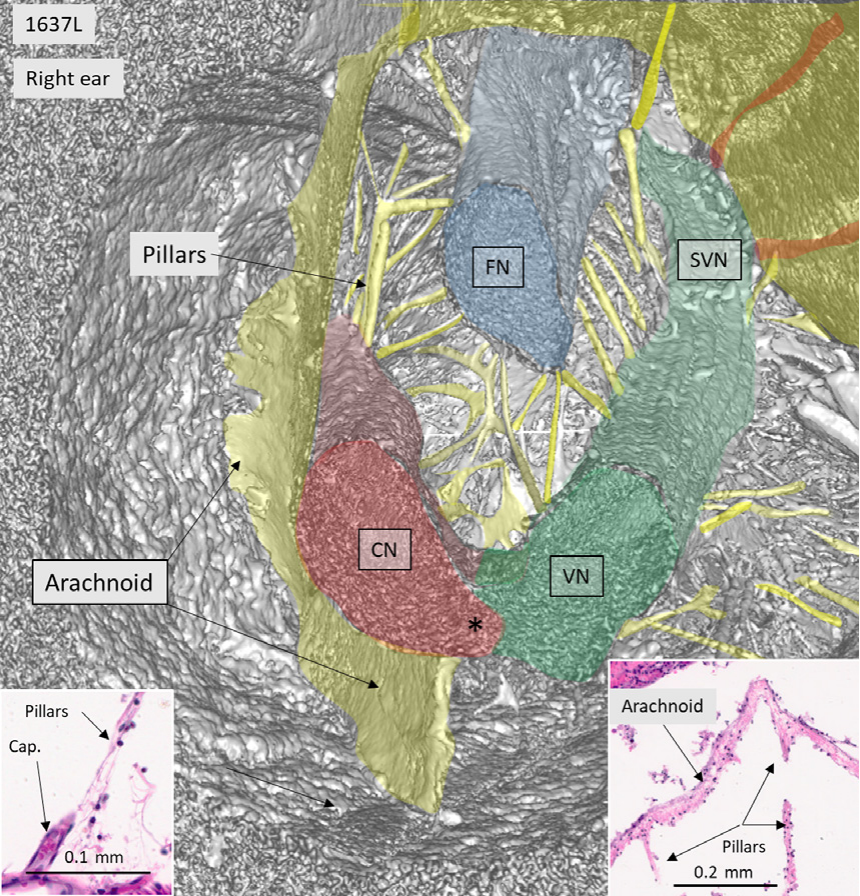
SR‐PCI and 3D reconstruction of IAC shown in Fig. 2, cross‐sectioned more medially. The cranial nerves with radiating pillars can be seen. Left and right insets represent H&E‐stained celloidin sections of a cross‐sectioned human IAC. The cistern wall consists of an arachnoid membrane surrounding the nerve complex. Pillars run between the nerves and the arachnoid and are occasionally associated with capillary vessels (left inset). Both the arachnoid and pillars consist of connective tissue fibers surrounded by fibrous cells. Histological sections were kindly provided by Dr Charles Liebermann, Massachusetts Eye and Ear Infirmary, Boston, MA, USA.

SR‐PCI reproduced the arachnoid and the acoustic‐facial cistern extending to the fundus of the IAC. It consisted of a thin, highly variable sheet surrounding the cochleo‐vestibulo‐facial nerve complex. The arachnoid was partly tagged onto the dura wall of the IAC, which was verified on the individual X‐ray section (Fig. 1, inset). Figure 2 shows the 3D anatomy of a cropped IAC with corresponding X‐ray section (Fig. 2, upper inset). The arachnoid is attached to the lateral wall of the IAC, but there are still wide subdural spaces located superiorly and inferiorly between the arachnoid and the bony wall. The cistern reaches the vertical crest (VC) where the superior vestibular nerve (SVN) and facial nerve (FN) enter the cistern. The arachnoid is seen to adhere tightly to the medial surface of the cochlear nerve (epineurium) and appears to suspend the CN via superior extension (Fig. 2, insets). Figure 3 shows the same IAC cropped more medially, with sustained connection between the CN and the VN. In this micrograph, a system of arachnoid pillars and trabeculae is outlined in the IAC cistern. Rods or pillars radiated circumferentially from the surface of the cranial nerves and, at times, to the bony wall of the IAC. In these cases, X‐ray scans disclosed that the arachnoid membrane was tagged to the inner wall of the IAC and that the pillars were always located within the cistern or subarachnoid space (Fig. 3). The pillars were impressive on 3D images after scalar opacity mapping but were challenging to envisage on individual scans. Histological sections obtained from the Laboratory in the USA verified their discrete existence. The pillars consisted of a central fibrous core surrounded by fibrocytic cells (Fig. 3, insets). The pillars and arachnoid contained blood vessels, mostly capillaries.

**Figure 4:**
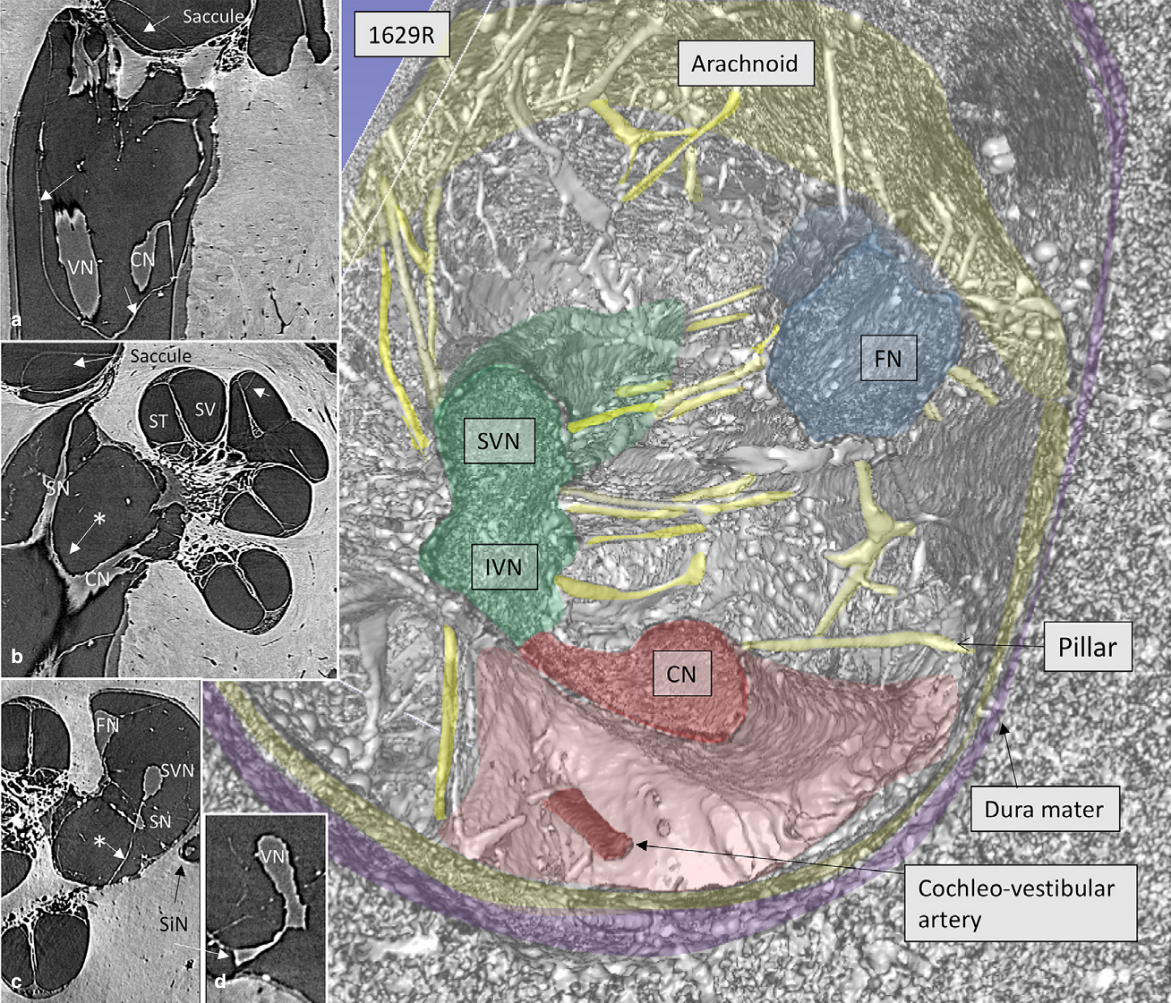
SR‐PCI and 3D reconstruction show the nerve complexes in a left IAC surrounded by a continuous arachnoid sheet (inset a). The arachnoid is firmly attached to the dura wall (stained purple) except superiorly, where there is a subdural space. Inset b shows a section with connection between the CN and SN (*) as well as arachnoid attachments to the IAC walls. In inset c, the arachnoid connections between the SVN and SN and fundus can be seen (*). The cochleo‐vestibular artery (CVA) enters among the high‐frequency nerve fibers and reaches the base of the cochlea. ST, scala tympani; SV, scala vestibule.

Figure 4 with insets show the nerve complex in an IAC surrounded by a continuous arachnoid firmly attached to the dura wall, except superiorly, where a subdural space is exposed. Connections between the CN and SN are observed. The arachnoid is tagged to the osseous wall of the fundus near the nerve margins of the foramina of the cranial nerves. The cochlear nerve with its ‘tail’ of high‐frequency fibers is contained by a thin ‘curtain’ of tissue similar to the arachnoid. The cochleo‐vestibular artery (CVA) enters among the high‐frequency nerve fibers and reaches the base of the cochlea.

**Figure 5:**
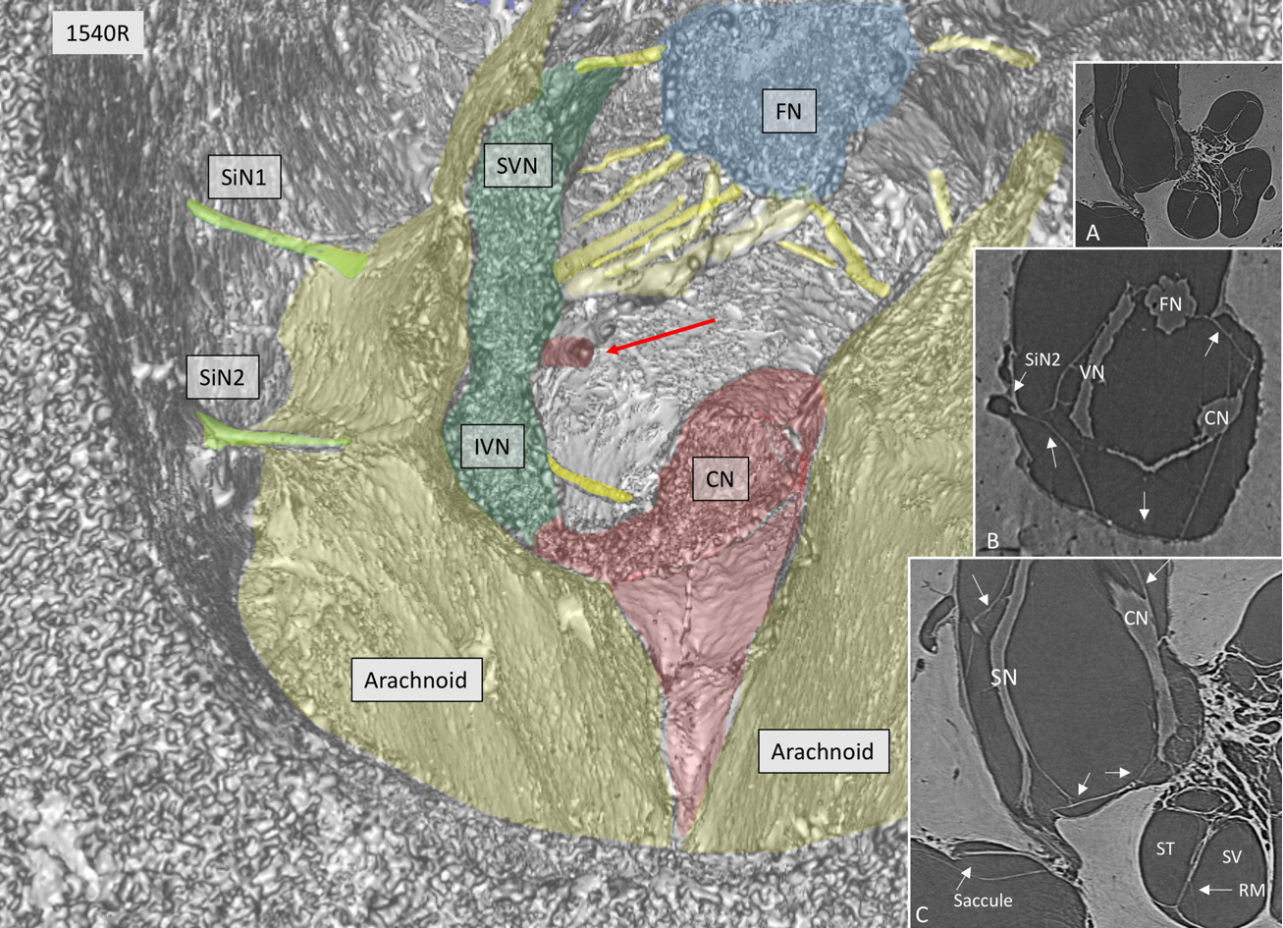
SR‐PCI of a left IAC showing the arachnoid cistern and cranial nerve complex. Two singular nerves (SiN 1, 2) exit from the vestibular nerve. The 3D image indicates a discontinuity at the floor of the IAC, but this is contradicted by sections showing continuous lining along the wall of the IAC (arrows in a and b). A blood vessel is seen near the vestibular nerve (red arrow). CN, cochlear nerve; RM, Reissner's membrane; SN, saccular nerve; ST, scala tympani; SV, scala vestibule.

At times, the arachnoid membrane was hardly visible at 3D, which was thought to be due to a limited optic resolution (Fig. 5). However, the elusive ‘rupture’ of the arachnoid depended on the tangential cropping and arachnoid adhesion to the IAC wall (Fig. 5, inset a). Figure 5 shows the arachnoid following the exiting singular nerve(s) to the bony orifice but leaves a space between the cistern and IAC bony wall (Fig. 5, inset b and c).

**Figure 6:**
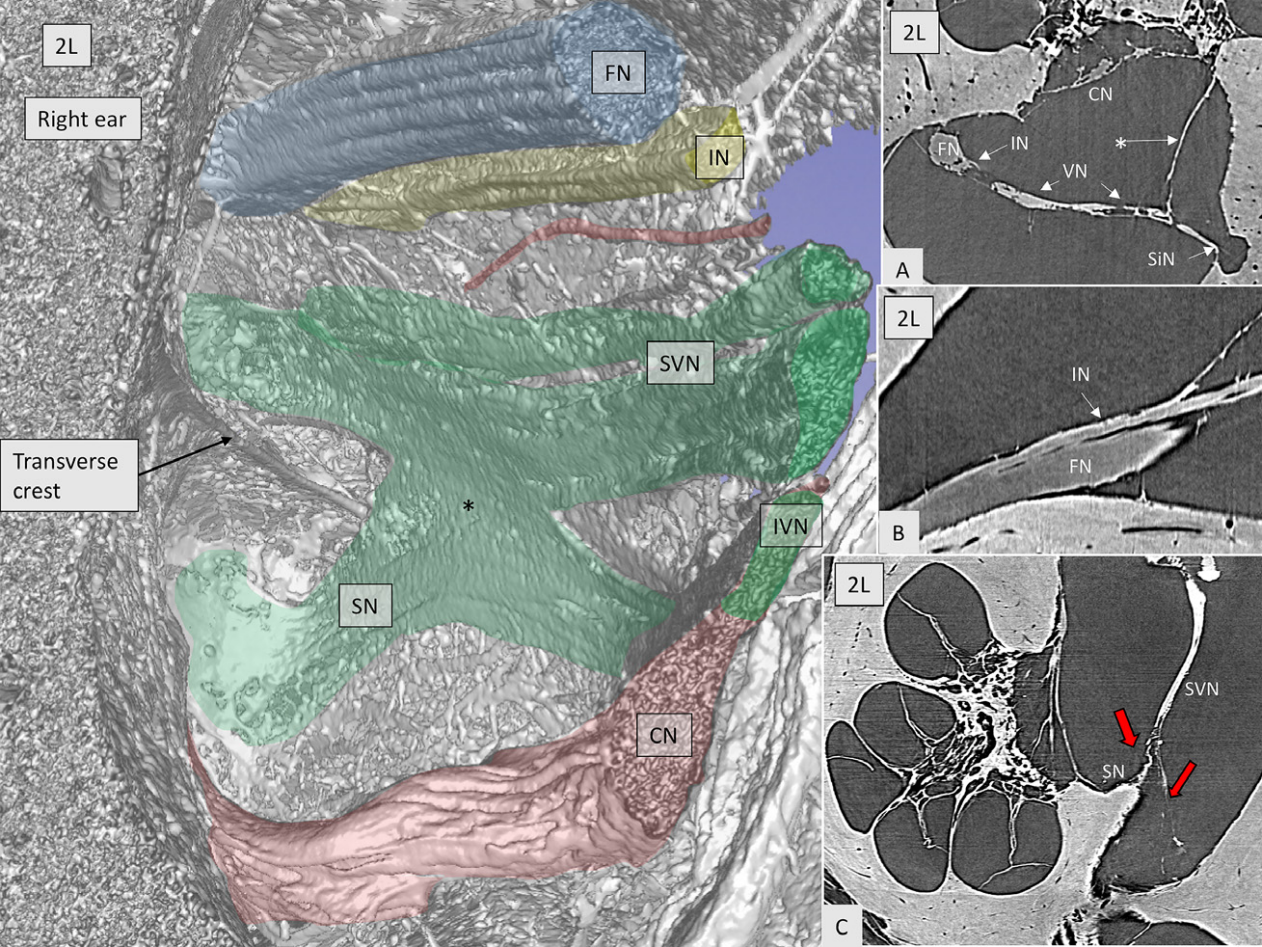
SR‐PCI imaging of the anastomoses between the inferior (saccular nerve, SN) and the superior vestibular nerves (*green, SVN) in a right ear. The intermediate nerve (yellow, IN) runs lateral to the facial nerve (FN). A blood vessel can be seen (red). Inset A shows the close connection between the vestibular nerve (VN) and the intermediate nerve (IN). * Arachnoid attachment. Inset B demonstrates the anastomosis between the IN and FN. In inset C, the SVN gives off a branch (left red arrow) to the SN before entering the superior vestibular nerve canal (right arrow in red).

**Figure 7:**
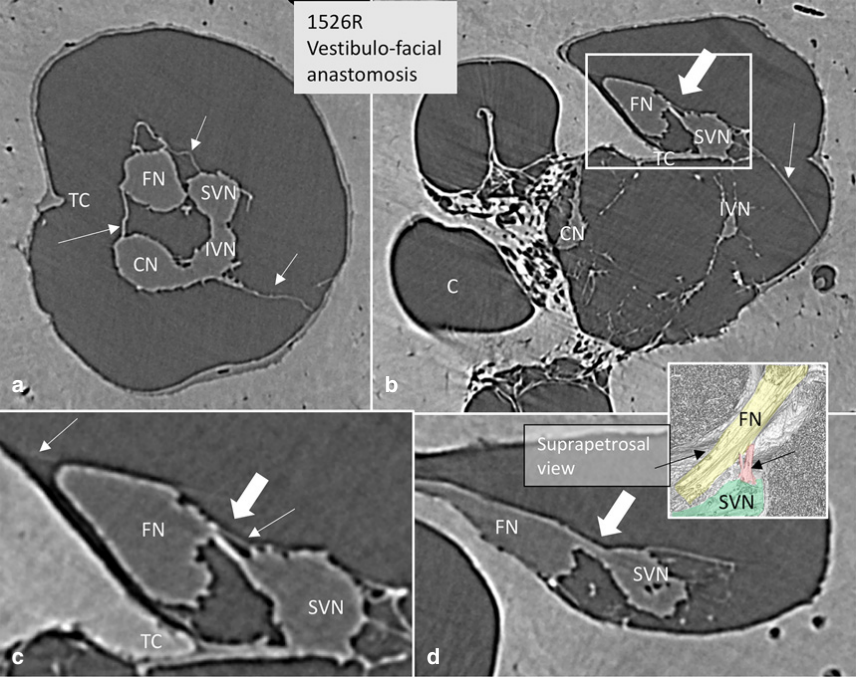
SR‐PCI of the vestibulo‐facial anastomosis. (a) Cross‐sectioned right IAC near the transverse crest (TC). The arachnoid folds are seen (arrows). (b) More lateral, anastomosing fibers exist (large arrow) between the SVN and the FN. Framed area is magnified in (c). (c) Framed area shows the nerve anastomosis (large arrow) and the arachnoid (thin arrows). There is no separation of arachnoid between the two nerves above the TC. (d) Connecting fibers (arrow) between the SVN and FN at the vertical crest running toward the FN. A similar arrangement can be seen in the colored inset.

Connections could be seen between the IVN (saccular nerve) and CN fibers directed to the base of the cochlea (Fig. 6). The SVN and IVN branches anastomosed before being separated by the transverse crest (TC). Nerve fibers coursed from the inferior to the superior branch and vice versa (Fig. 6, inset c). The intermediate nerve (IN) was identified in the fundus region running lateral to the FN where it connected to the FC (Fig. 6, inset b). The IN integrated entirely with the main trunk of the FN. In Fig. 6, inset b, the nerve anastomosis is seen at the level of the VC, a structure also named ‘Bill's bar’ by skull base surgeons. Horizontal sectioning also demonstrated the vestibulo‐facial anastomoses (Fig. 7). These fibers coursed in a distal direction from the SVN to the facial nerve.

**Figure 8:**
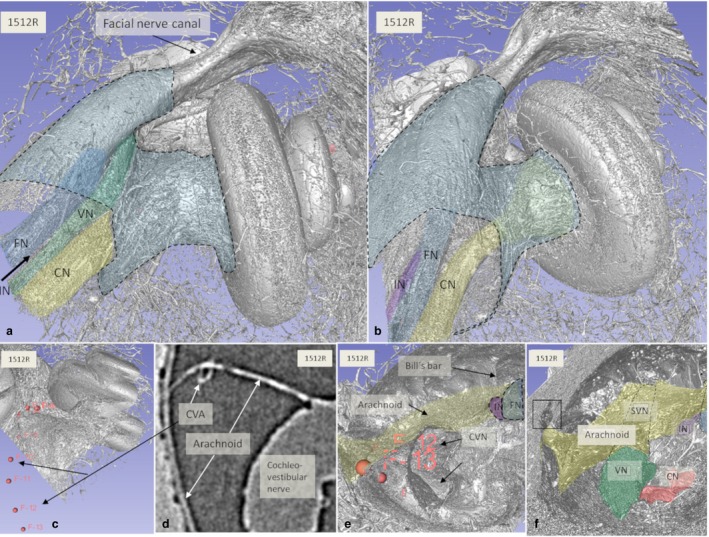
(a,b) SR‐PCI and 3D reconstructions of a left human cochlea [medial view; (b) slightly angled anti‐clockwise]. The medial part of the IAC (blue) and cranial nerves are seen. (c–e) The cochleo‐vestibular artery (CVA) could be traced from the arachnoid in the IAC to the cochlea by fiducial markers (c,e). Arachnoid tags the IAC wall inferiorly and follows the entrance of the singular nerve (frame in f). CVN, cochleo‐vestibular nerve; FN, facial nerve; IN, intermediate nerve; SVN, superior vestibular nerve; VN, vestibular nerve. Small arrow; TC.

Inner ear blood vessels running in the IAC were generally difficult to discern. Both the CVA and blood vessels along the vestibular nerves could be identified near the fundus in a few bones. The CVA perforated the curtain of tissue embracing the high‐frequency cochlear fibers (Fig. 4). One artery in the arachnoid of the IAC mid‐portion could be followed three‐dimensionally, using fiducial markers, to a tortuous artery in the base of the cochlea (Fig. 8a,b). It entered into the modiolus near the saccular nerve foramen (Fig. 8c–f). A foramen of the TC varied in size and joined either the saccule or the utricle. The canal housed a vessel, assumingly a branch of the anterior vestibular artery (AVA).

## Discussion

Recently, we analyzed the fundus region of the human IAC using micro‐CT (Schart‐Moren et al. 2017b). Bone channels, but not the soft tissues, could be reproduced. Here, we show that in‐line SR‐PCI can also reproduce the soft tissues. For the first time, the 3D anatomy of the human IAC, including cranial nerve complexes, anastomoses, arachnoid membranes, and invagination (acoustic‐facial cistern) in unprocessed, un‐decalcified specimens, could be reproduced. The cerebellum‐pontine cistern (Yasargil et al. 1976) formed a more or less continuous blind sac ending at the fundus region. The results substantiate earlier findings showing that vestibular schwannoma arising in the ganglionic area may grow unseparated from adjacent cranial nerves, without duplication of the arachnoid layers (Lescanne et al. 2002). These authors found that the arachnoid underlined the dura mater of the IAC from the acoustic meatus to the fundus. The subarachnoid space was termed the acoustic‐facial cistern, analogous to the trigeminal cistern or dura acoustic‐facial recess. The findings are consistent with earlier MRI studies showing that cerebrospinal fluid surrounds the nerve complexes (Held et al. 1997; Ryu et al. 1999). However, in some places, the arachnoid adhered to the surface of the nerve without an obvious subarachnoid space. This could be seen superiorly at the FN and SVN near the fundus and medially at the CN low‐frequency bundle (Fig. 2).

Our 3D study showed discontinuities of the arachnoid membrane, which were assumed to be caused by its fragility and postmortem changes occurring after removal of the brain. The thin arachnoid was at the limit of image resolution, and visualization was greatly dependent on computer adjustment with scalar opacity mapping and shading of the different bones scanned with various energy spectra. However, parallel views of sections showed that the arachnoid was continuous but tagged onto the dura wall of the IAC. Reduced intracranial pressure and postmortem changes may lead to separation of the arachnoid membrane from the dura and explain the variable size and anatomy of the cistern in the IAC.

The arachnoid ended above the TC, at the level of the entrances of the FN and SVNC where the nerves pierced the membrane wall. This arrangement ensured that CSF cannot escape or leak into the bony canals. The arachnoid enveloped the cranial nerves and vessels and on occasion fused with the dura lining of the wall of the IAC and the TC, creating separate cisterna ‘pockets’.

The singular nerve (SiN) innervating the posterior ampule entered the bony canal at variant distances from the fundus. This nerve can be used by the surgeon for anatomic guidance and nerve identification. After its separation, the IVN continues as the SN. Invariably, two SiNs entered separate channels (Fig. 5), verifying earlier observations by Bergstrom (1973), Gacek & Rasmussen (1961) and Montandon et al. (1970). The arachnoid extended around these nerves to the foramina.

A notable finding was the arachnoid pillars or villi radiating from the surfaces of the cranial nerves. They ran between cranial nerves, and between nerves and the IAC wall. They resembled the pillars and veil‐like structures or plates that form networks to provide mechanical support to neurovascular structures between the pia mater and arachnoid in the brain (Key & Retzius, 1876; Retzius, 1881; Yasargil et al. 1976; Mortazavi et al. 2018). The porous trabeculae consist of collagen I and are surrounded by fibroblast cells (Talbert, 2014). They dampen brain movement and reduce traumatic impact to the brain (Zoghi‐Moghadam & Sadegh, 2010) and the optic nerve (van der Rest & Garrone, 1991; Killer et al. 2003; Kierszenbaum, 2007), where they offer reinforcement and mechanical protection. The trabecular meshwork may be important for pressure homeostasis and dysregulation, such as in the optic nerve, where it may lead to optic neuropathy (Saboori & Sadegh, 2014). The pillars and trabeculae in the IAC could serve similar purposes, namely, to stabilize and protect individual cranial nerves and sustain their proper course. Amalgamation could lead to ephaptic nerve transmission and dysregulation. Such conditions might explain some cases of severe FN excitation after CI. At surgery of the IAC, retraction of the arachnoid may be avoided to maintain the integrity of the villi. The pillars and trabeculae could also be involved in local fluid regulation, thereby raising issues of their significance related to inner ear fluid homeostasis and dysfunction.

### Nerve anastomoses

Several nerve anastomoses are known to exist in the human IAC; these were also revealed with SR‐PCI and 3D reconstructions. The cochleo‐vestibular anastomosis (Oort) is between the saccular and CNs at the floor of the IAC. The nerve fibers direct to the basal turn of the cochlea (Oort, 1918; Hardy, 1934; Shute, 1951; Gacek & Rasmussen, 1961; Arnesen, 1984), which, once inside the cochlea, run spirally against the apex in the so‐called intra‐ganglionic spiral bundle. The bundle conveys efferent fibers from the olive in the brain stem (olivo‐cochlear bundle) together with some afferent fibers (Rasmussen, 1946).

The main nerve supply to the saccule is from the IVN, which runs beneath the TC (Retzius, 1881; Streeter, 1906; Voit, 1907; Oort, 1918; Hardy, 1934). Voit (1907) described contributions from the SVN as well, and this branch was named Voit's nerve. Arnesen (1984) & Bergstrom (1973) also described a nerve that coursed over the TC to reach the saccule macula. Micro‐CT showed that this nerve may enter an upper saccular nerve foramen (Schart‐Moren et al. 2017b). Synchrotron analyses verified this anastomosis between the SVN and IVN at the TC. Fibers ran from the SVN, reaching the SN and vice versa. Voit's nerve may, therefore, be derived from the SVN and reach the upper foramen of the SNC, as earlier described by Schart‐Moren et al. (2017b). The two saccular branches could be mistaken for a CN in MRI, and this finding may be clinically relevant in connection with CI.

Anastomoses between the facial and vestibular nerves are known to exist in the IAC (Paturet, 1951; House, 1961; Fisch, 1973; Silverstein, 1984; Rubinstein et al. 1996; Ryu et al. 1999; Ozdogmus et al. 2004), as well as more proximally through the intermedius nerve (Gacek & Rasmussen, 1961; Rhoton et al. 1968; Lescanne et al. 2002). According to Chouard (Chouard, 1975), the nerves are unseparated above the TC and are surrounded by the same pia sheath. These connections could be demonstrated with SR (Fig. 6b). The intermediate nerve was positioned lateral to the entrance of the FC where it integrated completely with the FN trunk. The direction of the connecting fibers was from the vestibular nerve to the FN. In some specimens, the intermediate nerve had fused with the FN more medially in the IAC. Therefore, it could not be specified whether the vestibulo‐facial anastomosis connected to the intermediate nerve. Chouard found them to be a few tenths of a mm thick and branched from the intermediate nerve to enter the vestibular nerve, generally proximal to Scarpa's ganglion but, in some cases, also distal to it. The connection was from the posterior edge of the intermediate nerve to the anterior edge of the upper vestibular nerve. Based on histological sectioning, he found evidence that these connections carry parasympathetic innervation. An etiological link to Meniere's disease was proposed, and removal, or vestibular neurectomy, of this acoustic‐facial anastomosis via a middle fossa approach resulted in improvement in hearing. Similar findings were described by Fisch (1970), Sterkers & Pietruski (1972), Portmann et al. (1969), and Fluur (1973). Vestibulo‐facial anastomoses between the SVN and FN in the most lateral portion of the IAC were demonstrated by Unel et al. (2012) using MRI. According to (Bergstrom, 1973), vestibulo‐facial anastomoses contain about 700 fibers. This study could not trace the source of blood vessels to the inner ear in the medial part of the IAC. However, vessels could be identified nearer the fundus region. A bone channel was described in the lateral part of the TC (Kozerska & Skrzat, 2015; Schart‐Moren et al. 2017b). It was thought to transmit blood vessels to the vestibule (V) or nerve branch and to the otolith organs. The present study verified that this channel contained a blood vessel, probably an artery, and most likely a branch of the anterior vestibular artery (AVA). The present study also identified and traced the CVA running in the arachnoid toward a foramen at the basal cochlea. Future modified staining techniques could further improve visualization of miniscule structures. Lareida et al. (2009) used a combination of osmium tetroxide staining and high‐resolution tomographic imaging with monochromatic X‐rays. This allowed visualization of cellular structures of the human inner ear, which was not possible here.

**Figure 9:**
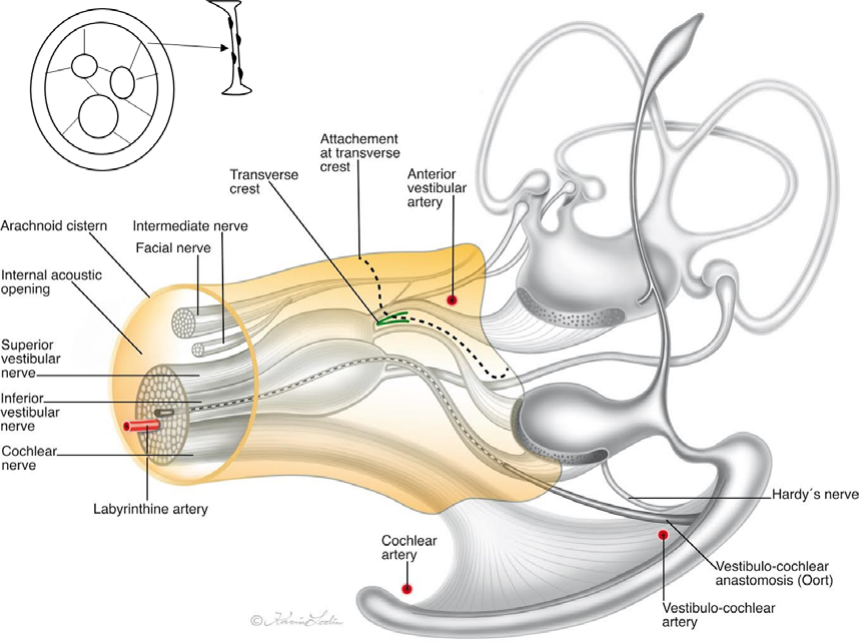
Illustration of the vestibulo‐cochlear‐facial nerve complex of the human IAC including the cistern and arachnoid surrounding as observed by SR‐PCI. Adapted partly from Oort (1918), de Burlet (1924), and Schart‐Moren et al. (2017). Inset shows the principal arrangement of arachnoid villi. Illustration made by Karin Lodin.

In brief, SR‐PCI identified the extension of the cerebellum‐pontine cistern into the fundus of the human IAC. The arachnoid wall of the ‘IAC cistern’ partly adhered to the cranial nerves at fundus. SR‐PCI verified results of earlier anatomic studies but added new 3D data on nerve anastomoses and blood supply (Fig. 9). The technique demonstrated a hitherto undescribed system of pillars or villi supporting the cranial nerves in the IAC. This system's functional significance and possible role in aural disease are discussed but will require additional study.

## Conclusions

SR‐PCI with 3D reconstructions was performed to analyze the complex anatomy of the cranial nerves and vessels in the acoustic‐facial cistern in the human IAC. Nerve anastomoses were verified. A system of arachnoid pillars and trabeculae were identified between the cranial nerves and arachnoid that were substantiated histologically. We conjecture that these structures support and functionally separate the cranial nerves, and may play a role in local fluid hydrodynamics. The present findings and descriptions may further assist in the surgical and radiological identification of anatomical structures in the human IAC. Novel techniques to image clinically the fine details of the inner ear with even higher precision are emerging.
